# Long Noncoding RNA RP11-357H14.17 Plays an Oncogene Role in Gastric Cancer by Activating ATF2 Signaling and Enhancing Treg Cells

**DOI:** 10.1155/2021/6635936

**Published:** 2021-05-29

**Authors:** Tang Xiaoli, Wang Wenting, Zhang Meixiang, Zuo Chunlei, Hu Chengxia

**Affiliations:** ^1^Department of Clinical Laboratory, The First People's Hospital of Lianyungang, Lianyungang, Jiangsu, China; ^2^Department of Clinical Laboratory, 2nd Affiliated Hospital of Nanjing Medical University, China; ^3^Department of Clinical Laboratory, Suqian Hospital of Traditional Chinese Medicine, Suqian, Jiangsu, China

## Abstract

**Background:**

Gastric cancer (GC) is one of the most common malignant tumors in the world. The potential functions and mechanisms of long noncoding RNAs (lncRNAs) in GC development are still unclear. It is of great significance to explore the prognostic value of LncRNA signatures for GC.

**Methods:**

LncRNAs differently expressed in GC and their prognostic value were studied based on The Cancer Genome Atlas (TCGA) database. The functional regulatory network and immune infiltration of RP11-357H14.17 were further studied using a variety of bioinformatics tools and databases.

**Results:**

We found that the high expression of RP11-357H14.17 was closely associated with shortened overall survival (OS) and poor prognosis in gastric cancer patients. We also found that its expression was related to clinical features including tumor volume, metastasis, and differentiation. Functional enrichment analysis revealed that RP11-357H14.17 is closely related to enhanced DNA replication and metabolism; ssGSEA analysis implied the oncogenic roles of RP11-357H14.17 was related to ATF2 signaling and Treg cell differentiation. Furthermore, we verified such link by using real-time PCR and IHC staining in human GC samples.

**Conclusion:**

We demonstrate that RP11-357H14.17 may play a crucial role in the occurrence, development, and malignant biological behavior of gastric cancer as a potential prognostic marker for gastric cancer.

## 1. Introduction

Gastric cancer (GC) is an invasive disease that continues to have a severe impact on global health [1]. Although its incidence has declined overall over the past few decades, GC is still ranked the fourth most common type of cancer and the second cancer-related deaths globally [1, 2]. Although the GC incidence has declined because of improved nutrition, better food preservation, better prevention, and earlier diagnosis and treatment, the prognosis still remains poor [1]. GC is usually diagnosed at an advanced stage. Gastric cancer is a heterogeneous disease, requiring continuous attention and research in prevention, early detection, and new treatment options.

Long noncoding RNAs (lncRNAs) are a group of noncoding RNAs to be defined as more than 200 bp in length with no protein-coding function [3]. LncRNAs were first discovered in 2002 in mice lacking of specific and complete open reading frames. After decades of researches, LncRNAs were proved to be an important transcriptional and translational regulator, involving in not only physiological but also pathological processes, including chromatin remodeling, transcription, posttranscriptional translation, cell proliferation, differentiation, and metabolic reprogramming. Abnormal expression of lncRNAs took part in the occurrence and development of a variety of malignancies, including prostate cancer [4], ovarian cancer [5], breast cancer [6], and gastric cancer [7]. The high expression of H19 [8], TUSC7 [9], MEG3 [10], and MALAT1 [11] is closely related to the poor prognosis of GC patients and promotes the proliferation, metastasis, and EMT of GC cells. These studies showed that lncRNA plays a crucial role in the prognosis and malignant biological behavior of GC.

In this study, real-time polymerase chain reaction (RT-PCR) was used to evaluate the expression level of RP11-357H14.17 in GC problems. We also assessed the relationship between RP11-357H14.17 levels and clinicopathological characteristics. Through bioinformatics analysis and experimental verification, its role in GC was predicted and analyzed. Our findings may contribute to a better understanding of the role of RP11-357H14.17 as a regulatory factor for GC and as a possible candidate target for new diagnosis and treatment.

## 2. Materials and Methods

### 2.1. Bioinformatics Analysis

The software R package limma (V.3.40.6) was used to calculate the fold-change of transcripts and to screen for differentially expressed genes (DEGs) in the RNA-seq cohort. A fold-change larger than two and an adjusted *p* value less than 0.05 were set as the cut-off values for screening significant DEGs. Cluster analysis and heatmap generation were performed using the R package clusterProfiler (V.3.12.0) and pheatmap (V.1.0.12), respectively. 23 Gene Ontology (GO) and Kyoto Encyclopedia of Genes and Genomes (KEGG) pathway analyses for the dysregulated genes were performed by ClueGO25 (V.2.5.5), within Cytoscape (V.3.7.1). A false-discovery rate less than 0.05 was set as the cut-off value.

### 2.2. The Prognosis of Dysregulated lncRNAs Analyzed with GEPIA and Kaplan-Meier Plotter

GEPIA (http://gepia.cancer-pku.cn/) [12] is a newly web-based tool that contains sequencing expression data from 9736 tumor samples of 33 cancer types and 8587 normal samples. The database includes a variety of analysis modules such as differential gene expression analysis, survival and prognosis analysis, correlation analysis, and dimensionality reduction analysis. In this study, GEPIA database was employed to further analyze the expression and prognostic value of differentially expressed lncRNAs in STAD. The expression analysis of these genes performed by one-way ANOVA, and the filter criteria were as follows: ∣Log2FC | >1, *p* value < 0.05, “median,” hazards ratio (HR), and 95% confidence interval. The Kaplan-Meier (KM) Plotter (http://kmplot.com) is an effective tool for detecting the prognosis of patients with tumors. According to the expression of lncRNAs, patients with EC were divided into two groups: high and low expression groups. The hazard ratio (HR) at a 95% confidence interval and log-rank *p* values were also investigated online. The filter conditions were as follows: cancer: pan-cancer RNA-seq (Uterus corpus endometrial carcinoma); survival: overall survival (OS); follow-up threshold: 120 months.

#### 2.2.1. The Cellular Localization of lncRNAs

UCSC (https://genome-asia.ucsc.eduk/index.html) provides a web-based interface to help users browse the genetic information, view the genome annotation assembly, and download gene sequences. LNCipedia (https://lncipedia.org) is a free human lncRNA transcription sequence and structure annotation database. LncLocator (https://LncLocatorwww.csbio.sjtu.edu.cn/bioinf/lncLocator/) is based on integrated classifier to predict lncrna subcellular localization of free public platform. Only by using the sequence information of lncRNA, the distribution ratio of lncRNA in cytoplasm, nucleus, ribosome, cytoplasm, and exosome can be obtained rapidly. In this study, sequence information of lncRNAs was detected by UCSC and LNCipedia, and cellular localization of lncRNAs was determined by LncLocator.

#### 2.2.2. Real-Time PCR

Total RNA in GC tissues was isolated using TRIzol reagent. The expression of genes of interest was detected using the syber-green-based real-time PCR. The primers for genes used in the study were listed in Table 1.

#### 2.2.3. Patients

The hospital-based case-control study consists of 52 patients newly diagnosed with gastric cancer. All the subjects were recruited from the 1st People's Hospital of Lianyungang and Suqian Hospital of Chinese traditional medicine, between February 2016 and August 2019. Patients with other hematological disorders, previous history of cancers, radiotherapy, and chemotherapy were excluded. The cancer-free control subjects from the same geographic area showed no evidence of a genetic relationship with the cases. The patients were classified according to World Health Organization classification. This study was approved by the Institutional Review Board of the 1st People's Hospital of Lianyungang and Suqian Hospital of Chinese traditional medicine (LL-16-12 and SCY-17-15), and every patient had written informed consent. The clinical features of all the cases and controls were presented in Table 2.

#### 2.2.4. IHC Staining

Sections were stained according to the previous publication. The section was incubated within primary mouse anti-human Ab for ATF2(ab239361), WT1(ab89901), CD4(ab183685), Foxp3(ab215206), and TGF-*β*(ab215715); the sections were stained with DAB according to manufacturer's protocols and mounted and photographed using a digitalized microscope camera (Nikon, Japan).

## 3. Results

### 3.1. RP11-357H14.17 Is Overexpressed in Human Stomach Cancer

**Figure 1:**
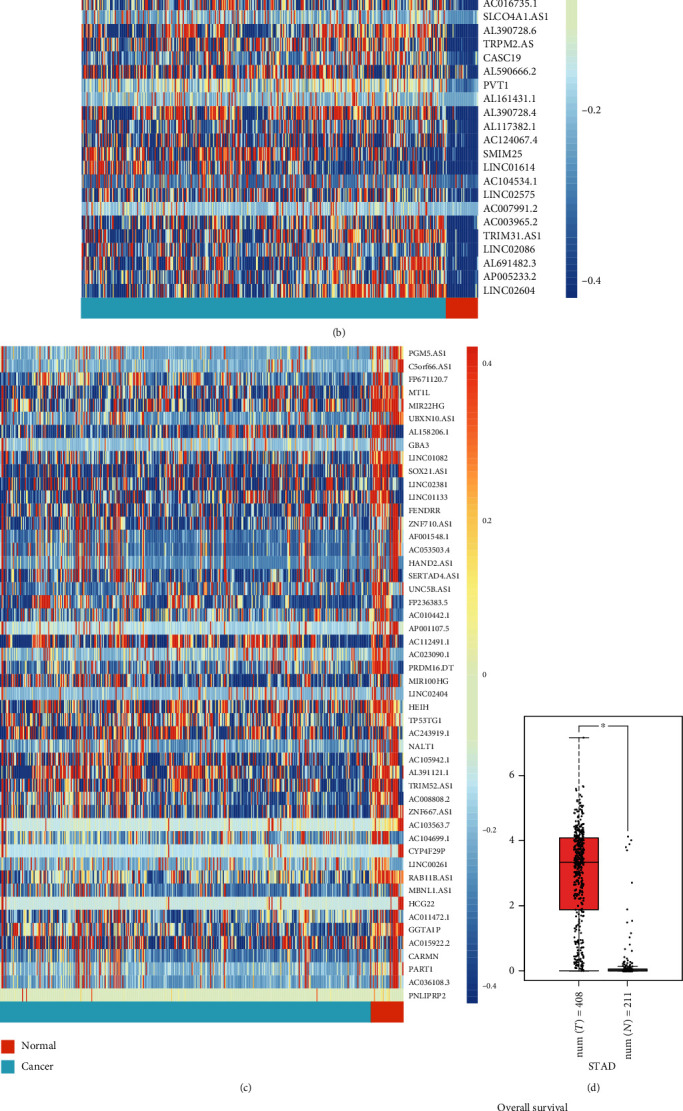
RP11-357H14.17 is overexpressed in human stomach cancer. (a) Heatmap of the all significantly differently expression lncRNAs (*n* = 3790, cancer vs. normal, adj.*p* < 0.05). (b, c) Heatmaps of TOP 50 up and down-regulated significantly differently expressed lncRNAs; RP11-357H14.17 was highlighted with red font in (b). For (a–c), rows denote lncRNAs, and columns represent patients. Blue to red indicates a trend from low to high expression. Boxplot of comparison between cancer and normal control tissues (d) and cancer tissues with different TMN stages (e) in RP11-357H14.17 expression; the data is provided by TCGA-STAD. (f) Comparison of overall survival between RP11-357H14.17 high expression and low expression group; the data is provided by TCGA-STAD. (g) Boxplot of comparison between cancer and normal control tissues (left) and cancer tissues with different TMN stages for 52 paired human GC tissues (right). ^∗∗∗∗^*p* < 0.0001, for the left panel, unpaired *t* test, and for the right panel, one-way ANOVA. (h) Prediction of subcellular location of RP11-357H14.17 provided by online tool lncLocator.

To fully screen the long noncoding RNA within human stomach cancer, we analyzed total 18036 lncRNAs within the Cancer Genome Atlas Stomach Adenocarcinoma (TCGA-STAD) data collection; we found 375 GC patients and 32 normal controls with the TCGA-STAD. All of the 18036 lncRNAs were compared within the GC patients and normal controls by using Limma R package; the results indicated that 3790 lncRNAs were significantly differently expressed (adjusted *p* < 0.05) within these two groups, including 1642 down-regulated and 2148 up-regulated (Figure 1(a)). We then rearranged the up-regulated and down-regulated lncRNAs by logFc in decreasing order; we listed top 50 up- and down-regulated lncRNAs in Figures 1(b) and 1(c). Within these 100 lncRNAs, RP11-357H14.17 was the most up-regulated lncRNAs.

RP11-357H14.17 is located in chromesome 17 with a Ensembl ID of ENSG00000272763.1. We confirmed its expression in human GC tissues by using an online tool, Gepia [12]. By involving the samples from the Genotype-Tissue Expression (GTEx) and TCGA-STAD (408 tumor samples and 211 normal control), we found that RP11-357H14.17 is overexpressed in human stomach cancer (Figure 1(d)). However, there is no significant difference in RP11-357H14.17 expression with different TMN stage (Figure 1(e)). The effect of RP11-357H14.17 on overall survival (OR) of GC patients was also analyzed which implied that high expression of RP11-357H14.17 was associated with poor prognosis of GC patients (*p* = 0.044) (Figure 1(f)). Furthermore, 52 paired human GC samples were collected for our study; we found that RP11-357H14.17 was overexpressed in GC tissues compared to paired adjacent tissues (Figure 1(g)). Moreover, we found that GC tissues with higher TMN stage was associated with high expression of RP11-357H14.17 (Figure 1(g)). The subcellular localization of RP11-357H14.17 was predicted by using an online tool: lncLocator; the result indicated that RP11-357H14.17 was mainly located in cytosol (36%) and ribosome (30%) (Figure 1(h)). The expression of RP11-357H14.17 was also significantly associated with poor histological differentiation (Table 2).

### 3.2. Increased Expression of RP11-357H14.17 Is Associated with Increased DNA Replication and Metabolism in Human Gastric Cancer

**Figure 2:**
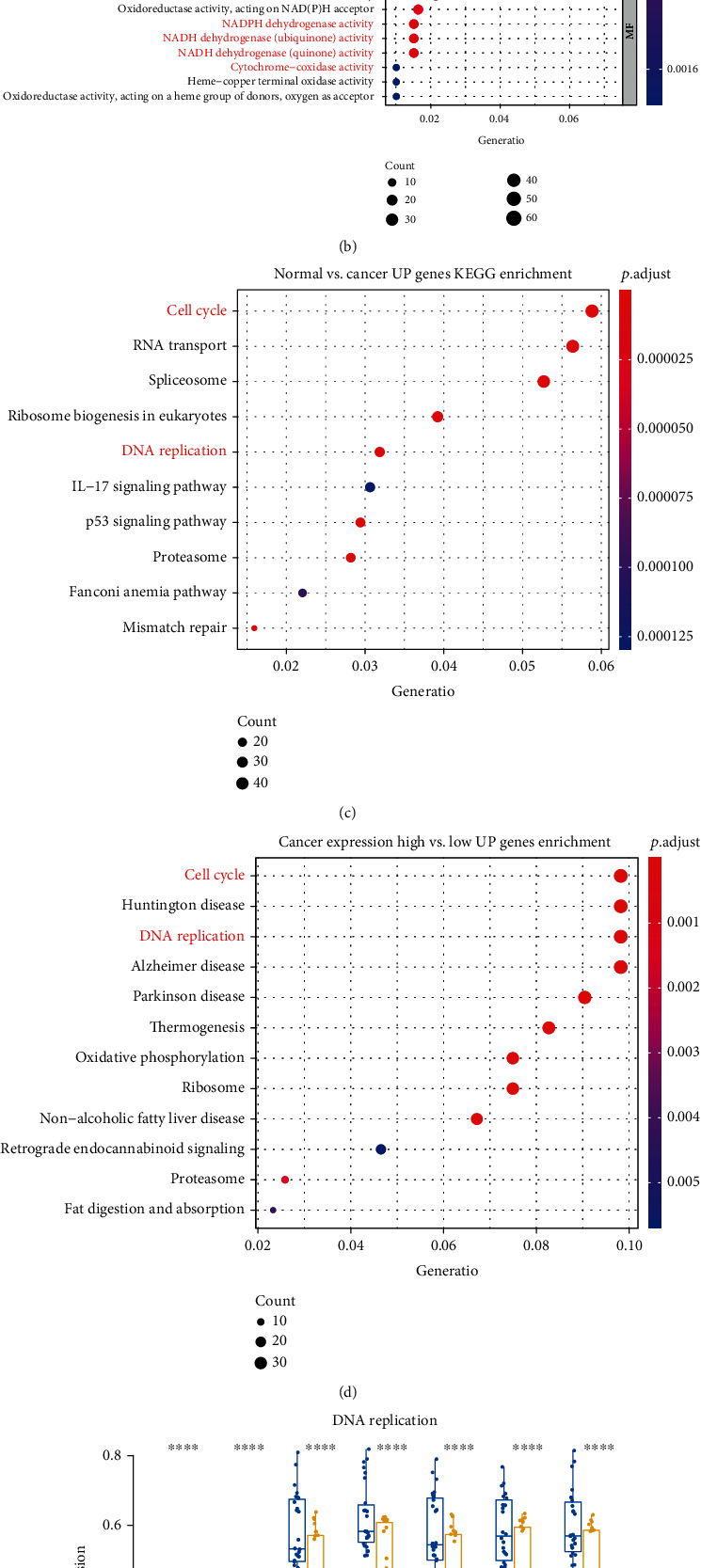
Increased expression of RP11-357H14.17 is associated with increased DNA replication and metabolism in human gastric cancer. GO enrichment was performed using significantly up-regulated genes compared between cancer and normal controls (a) and RP11-357H14.17 high and low expression sample sets (b). KEGG enrichment was performed using significantly up-regulated genes compared between cancer and normal controls (c) and RP11-357H14.17 high and low expression samples from TCGA-STAD (d). Representative genes expression stands for “DNA replication” (e) and “NADPH dehydrogenase activity” (f). GO terms was determined by real-time PCR. ^∗∗∗∗^*p* < 0.0001, one-way ANOVA.

To further investigate the oncogenic roles of RP11-357H14.17 in human GC, first, we performed KEGG and GO enrichment analysis using up-regulated DEGs obtained by two comparison patterns. One is the comparison between RP11-357H14.17 top-32 expression GC samples and normal GC control samples (T vs. N), and the other is the comparison between the GC samples with top 40 and bottom 40 RP11-357H14.17 expression (High40 vs. Low40). We figured out shared GO enrichment terms between two comparison described above including “DNA replication,” “nuclear DNA replication” in Biological Processes (BP) GO terms, “mitochondrial protein complex,” “respiratory chain” in the Cell Components (CC) GO terms, and “electron transfer activity” and “NADPH dehydrogenase activity” in Molecular Functions (MF) GO terms (Figures 2(a) and 2(b)). For KEGG enrichment, we found shared terms including “cell cycle” and “DNA replication” which implied similar events as GO enrichment (Figures 2(c) and 2(d)). These results might suggested that the overexpression of RP11-357H14.17 might seriously related to increased DNA replication and metabolism in human GC cells. In order to further confirm the shared terms implied by GO and KEGG enrichment, we selected a set of genes including “*RECQL4*, *ATAD5*, *GINS1*, *ORC6*, *ARD51*, *EME1*, and *CCND1*” for the GO term “DNA replication,” and “HMGCR, DHFR, FASN, DECR1, DUS2, GRHPR, and MTRR” for the GO term “NADPH dehydrogenase activity” using 52 paired human GC samples. We found that these genes were highest in those RP11-357H14.17 higher expression GC samples, significantly lower in the RP11-357H14.17 lower expression GC samples, and lowest in adjacent normal tissues (Figures 2(e) and 2(f)).

### 3.3. The Oncogenic Roles of RP11-357H14.17 Was Partially via Promoting ATF2 Signaling in Human Gastric Cancer

**Figure 3:**
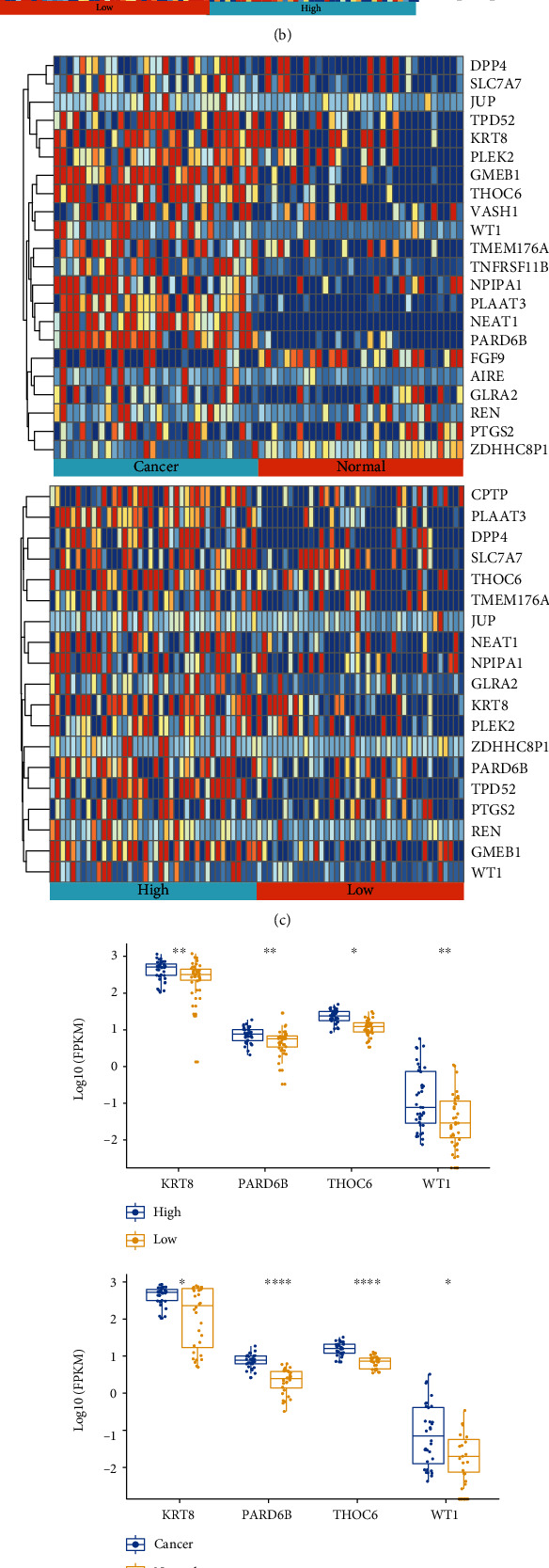
RP11-357H14.17 promotes the malignancy by promoting ATF2 signaling in human gastric cancer. Heatmap of all significantly different gene sets of c6 ssGSEA analysis for comparison between cancer and normal control samples (a) and RP11-357H14.17 high and low expression samples (b) from TCGA-STAD. ATF2-related gene sets were highlighted with red font (c). Heatmap of up-regulated genes in “ATF2_up.v1_DN” gene set derived from comparison between cancer and normal control samples and RP11-357H14.17 high and low expression samples from TCGA-STAD. The representative genes stand for “ATF2_up.v1_DN” gene set were highlighted in bold. (d) Representative gene expression stands for “ATF2_up.v1_DN” gene set was compared between cancer and normal control samples, RP11-357H14.17 high and low expression samples from TCGA-STAD, ^∗^*p* < 0.05, ^∗∗^*p* < 0.01, and ^∗∗∗∗^*p* < 0.0001, by unpaired *t* test, and 52 paired GC tissues categorized as RP11-357H14.17 high, low expression, and normal control, ^∗∗∗∗^*p* < 0.0001, one-way ANOVA (e). (f) Representative IHC staining figures for ATF2 and WT1 protein and comparison between 52 paired GC tissues categorized as RP11-357H14.17 high, low expression, and normal control, ^∗∗∗∗^*p* < 0.0001, one-way ANOVA.

In order to further clarify the possible mechanism for RP11-357H14.17-associated malignancy, we did single sample GSEA (ssGSEA) analysis using oncogenic signatures (c6) gene sets using “N vs. T” and “High 40 vs. Low40”. We found that high expression of RP11-357H14.17 in stomach cancer tissues were associated with ATF2-related gene sets including “ATF2_s_UP v1_up,” “ATF2_up. V1_up,” and “ATF2_up.V1_DN” (Figures 3(a) and 3(b)). We picked four genes including *KRT8*, *PARD6B*, *THOC6*, and *WT1* which were involved in all three gene sets. Their expressions were confirmed in TCGA-STAD data indicating that RP11-357H14.17 high expression samples have significantly higher expression of these four genes compared to RP11-357H14.17 low expression samples and normal control samples (Figures 3(c) and 3(d)). Next, we also investigated the expression of these four genes in 52 paired human GC samples; we found that all these four genes were significantly overexpressed in RP11-357H14.17 high expression samples and significantly lower in RP11-357H14.17 low expression samples and paired normal control samples (Figure 3(e)). Lastly, we analyzed the protein expression of ATF2 and WT1 in the 52 paired human GC samples; the result was consistent to the results obtained from real-time PCR; WT1 and ATF2 were significantly higher in RP11-357H14.17 high expression samples compared to RP11-357H14.17 low expression samples and paired normal control samples (Figure 3(f)).

### 3.4. RP11-357H14.17 Promotes GC Development by Inducing Treg-Associated Immunosuppression

**Figure 4:**
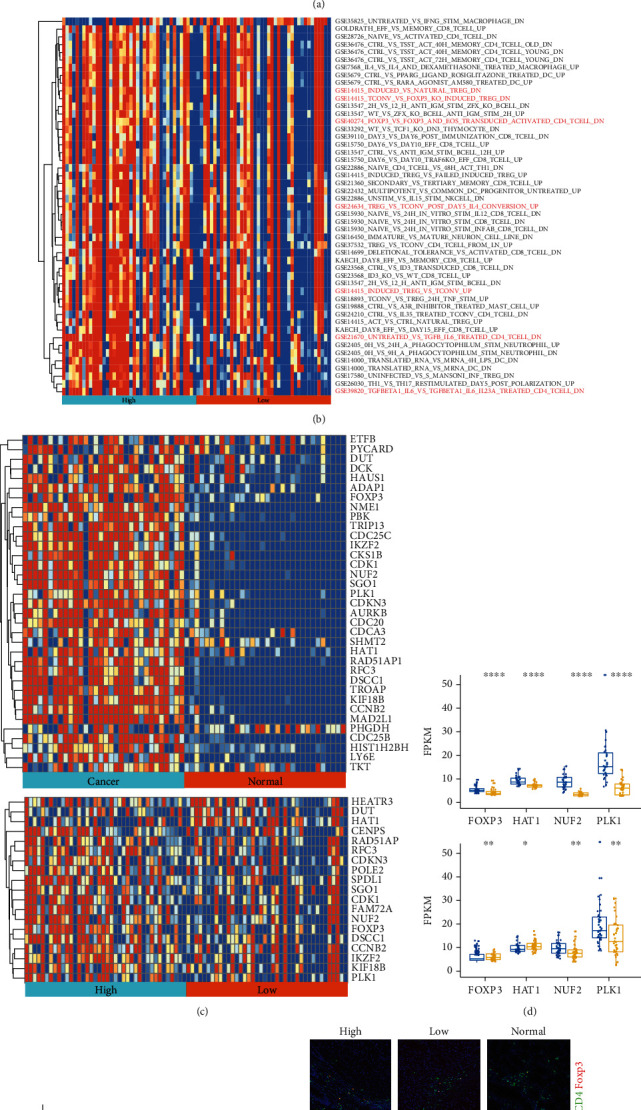
RP11-357H14.17 promotes the immunosuppression in human gastric cancer tissues by increasing Treg cell percentage. Heatmap of all significantly different gene sets of c7 ssGSEA analysis for comparison between cancer and normal control samples (a) and RP11-357H14.17 high and low expression samples (b) from TCGA-STAD. The “Treg” cell-related gene sets were highlighted in red font. (c) Heatmap of up-regulated genes in “GSE37532_TREG_VS_TCONV_CD4_TCELL_FROM_UP” gene set derived from comparison between cancer and normal control samples and RP11-357H14.17 high and low expression samples from TCGA-STAD, and the representative genes were highlighted in bold. (d) Representative gene expression stands for “GSE37532_TREG_VS_TCONV_CD4_TCELL_FROM_UP” gene set was compared between cancer and normal control samples, RP11-357H14.17 high and low expression samples from TCGA-STAD, ^∗^*p* < 0.05, ^∗∗^*p* < 0.01, and ^∗∗∗∗^*p* < 0.0001, by unpaired *t* test, and 52 paired GC tissues categorized as RP11-357H14.17 high, low expression, and normal control, ^∗∗∗∗^*p* < 0.0001, one-way ANOVA (e). (f) Representative multiple-color IHC staining figures for CD4, Foxp3, and TGF-*β* protein and comparison between 52 paired GC tissues categorized as RP11-357H14.17 high, low expression, and normal control. ^∗∗∗∗^*p* < 0.0001, one-way ANOVA.

To study the role of RP11-357H14.17 in tumor microenvironment (TME), we analyzed the immune-related roles of RP11-357H14.17 by ssGSEA analysis using immunologic signatures (c7) including 4872 gene sets for two sample sets described above. The results from both comparisons indicated that RP11-357H14.17 was significantly associated with Treg cell percentage within human GC by sharing gene sets “GSE37532_TREG_VS_TCONV_CD4_TCELL_FROM_LN_UP,” “GSE24634_TREG_VS_TCONV_POST_DAY5_IL4_CONVERSION_UP,” “GSE18893_TCONV_VS_TREG_24H_TNF_STIM_UP,” “GSE14415_TCONV_VS_FOXP3_KO_INDUCED_TREG_DN,” and “GSE14415_NATURAL_TREG_VS_TCONV_DN” (Figures 4(a) and 4(b)). Next, we have chosen one of the gene set “GSE37532_TREG_VS_TCONV_CD4_TCELL_FROM_LN_UP” and grip its involving genes from the TCGA-STAD database. The results were presented in Figure 4(c); four genes including FOXP3, PLK1, and HAT1 NUF2 which all reported to be related to Treg cell differentiation were selected; their expression was analyzed in both the TCGA-STAD database and 52 paired human GC samples. We found the four Treg cell-related genes were highest in RP11-357H14.17 high expression GC samples, lower in RP11-357H14.17 low expression GC samples, and lowest in normal control samples (Figures 4(c) and 4(d)). Last, we detected Treg cells percentage as well as the immunosuppression effector, TGF-*β* in human GC samples by using multiple-color IHC staining; we found that Treg cells were most abundant in RP11-357H14.17 high expression tissues, relatively lower in RP11-357H14.17 low expression tissues, and lowest in adjacently normal tissues (Figure 4(e)).

## 4. Discussion

LncRNA significantly regulates gene expression in both nucleus and cytoplasm [3]. In the nucleus, lncRNA binds to the Polycomb Group protein (PcG) complex to induce histone trimethylation and regulate mRNA expression of related genes at the transcriptional level [13]. At the same time, lncRNAs directly bind to promoters and regulate gene expression [14]. In cytoplasm, lncRNA also binds directly to mRNA, affecting its stability and expression at the transcriptional level [3]. Meanwhile, cytoplasmic lncRNAs can regulate gene expression in a posttranscriptional level. As previously mentioned, lncRNAs, together with miRNAs, act as sponges and induce a “ceRNA” to regulate gene expression [3]. On the other hand, lncRNA also affects the stability of proteins and inhibits their expression at the posttranscriptional level [13]. In the present study, by analyzing the TCGA-STAD data, we found that RP11-357H14.17 is the most up-regulated within human gastric cancer. Also, through an online predicting tool, lncLocator, we found that RP11-357H14.17 was located in cytosol and ribosome, which implied that most likely RP11-357H14.17 can be affected by sponges miRNA to regulated certain gene. So far, only two researches implied the role of RP11-357H14.17 in human cancers [15, 16]. One of the researches is concerning its role in diffuse-type gastric carcinoma (DGC). Overexpression of RP11–357H14.17 was spotted in human DGC; its expression were associated with increased tumor size, deeper depth of invasion, lymphatic metastasis, and advanced pathological stage [15]; this results was similar to our finding.

By using ssGSEA on c6 gene sets, we found that RP11-357H14.17 was associated with enhanced ATF2 signaling. Activated transcription factor 2 (ATF2) is a member of the activated protein 1 (AP-1) transcription factor family that contains DNA-binding proteins in basic Leucine Zipper (bZIP) [17, 18]. The N-terminal zinc finger region and the deactivation region activate the transcriptional activity of ATF2, while the C-terminal tissue has homologous dimerization and heterodimerization [19]. Its heterodimer partners are JUN, FOS, CREB, and MAF, leading to the formation of the AP-1 transcription factor [17]. ATF2 can also act as an epigenetic regulator, acting as a histone acetyltransferase (HAT), specifically acetylating histones H2B and H4, and triggering its own DNA binding effect [17]. The transcriptional functions of ATF2 show a wide range of target genes, which can be grouped into the following categories including cell cycle, immune and inflammatory responses, AP-1 binding partner, and apoptosis [19]. Our results also indicated that ATF downstream genes including *KRT8*, *PARD6B*, *THOC6*, and *WT1* were well correlated to RP11-357H14.17 expression. The detailed mechanism is worthy to be further explored in the future. Besides the regulation by ATF2 on the genes indicated above, a previous study revealed that anisomycin, a potent activator of ATF2, and JNK, induces expression of FoxP3 in both normal and malignant mammary epithelial cells [20]. This result might also implied that ATF2 activation in RP11-357H14.17 high expression GC tissues might also be related to increased Treg cell percentage.

By using ssGSEA on c7 gene sets, we found the high expression of RP11-357H14.17 was also associated with Treg cell-related immunosuppression, which can also be the other reason for RP11-357H14.17-related tumorigenesis. Regulatory T cells (Tregs) are a specific subset of T cells whose role is to suppress the immune response, thereby maintaining homeostasis and self-tolerance [21]. Studies have shown that Treg can inhibit T cell proliferation and cytokine production and play an important role in preventing autoimmunity [21].

In summary, by using bioinformatic analysis, we found that RP11-357H14.17 was overexpressed within human gastric cancer tissues. High expression of RP11-357H14.17 was associated with higher TMN stage, poor prognosis, and poor differentiation by promoting DNA replication. Deeper analysis revealed that RP11-357H14.17 was associated through ATF2 signaling and enhanced immunosuppression by promoting Treg cell percentage within human GC tissues.

## Figures and Tables

**Table 1:** Primer sequence for genes of interest.

Gene	Primer (forward/reverse)
RP11-357H14.17	TCCGGCCATCTGGCGCT
	CAAAGGCGACGTGCCGG
RECQL4	GCGCTCTACCGGGAATACC
	CAGCCCGATTCAGATGGGG
ATAD5	GTGAAGGACTGCGAGATTGAG
	TGTCTCTAGTCTTCCCTAGTGGT
GINS1	ACGAGGATGGACTCAGACAAG
	TGCAGCGTCGATTTCTTAACA
ORC6	ACAAGGAGACATATCAGAGCTGT
	AGTGGCCTGGATAAGTCAAGAT
RAD51	CCTCCTCTTTAACGCCTCCTG
	GGGGACAACTCCCAGACTTTTT
EME1	TCTGAGGAGTTGCCAACATTTG
	GGCTTCACAATCTGAGATGTCAA
CCND1	GCTGCGAAGTGGAAACCATC
	CCTCCTTCTGCACACATTTGAA
DBF4	CCGGAAAGTCCTTTTACTTGGAT
	AACCCTCAATTACCCCACCCA
CDC25C	TCTACGGAACTCTTCTCATCCAC
	TCCAGGAGCAGGTTTAACATTTT
MCM2	ATGGCGGAATCATCGGAATCC
	GGTGAGGGCATCAGTACGC
NASP	AGATTGGGAACCTAGAGCTTGC
	ACTTCTCCGAGTTTAAGATGTGC
EZH2	AATCAGAGTACATGCGACTGAGA
	GCTGTATCCTTCGCTGTTTCC
PCNA	CCTGCTGGGATATTAGCTCCA
	CAGCGGTAGGTGTCGAAGC
CDC25A	GTGAAGGCGCTATTTGGCG
	TGGTTGCTCATAATCACTGCC
KRT8	TGTGCCTACCTGCGGAAATC
	CTATGACCGAGGTGTCTGAGA
PARD6B	TTGGAGCTGAATTTCGTCGGT
	AGCCTACCAAAACGTCAACATT
THOC6	TCCCAGAGCGTCTCACCAT
	CCACCGGCTTCTTACTTTCCT
WT1	ACCAAAAATCGGTTCTTCATCCC
	TCACATCACTGGCAATCTTAGGA
ATF2	CACCAGCAGCACGAGTCTC
	TGTGCGAGGCAAACAGGAG
FOXP3	GTGGCCCGGATGTGAGAAG
	GGAGCCCTTGTCGGATGATG
HAT1	AAGCCATTCGGAACCTTACTTC
	AGTGCCATCTTTCATCATCCAC
NUF2	GGAAGGCTTCTTACCATTCAGC
	GACTTGTCCGTTTTGCTTTTGG
PLK1	CAGTCACTCTCCGCGACAC
	GAGTAGCCGAATTGCTGCTG

**Table 2:** Frequency distributions of selected variables in gastric cancer patients.

Features	RP11-357H14.17^High^		RP11-357H14.17^High^		*p*
	*N*	%	*N*	%	
Age (years)					0.779
≤50	10	19.23%	12	23.08%	
>50	16	30.77%	14	26.92%	
Gender					0.999
Male	14	26.92%	13	25.00%	
Female	12	23.08%	13	25.00%	
Differentiation					0.0254
G1-G2	17	32.69%	8	15.38%	
G3-G4	9	17.31%	18	34.62%	
Tumor size (cm)					0.0145
≤5 cm	16	30.77%	7	13.46%	
>5 cm	10	19.23%	19	36.54%	
TMN stage					0.0118
I-II	18	34.62%	8	23.08%	
III-IV	8	15.38%	18	26.92%	
*H*. *pylori* infection					0.999
Positive	16	30.77%	15	28.85%	
Negative	10	19.23%	11	21.15%	

## Data Availability

The RNA-seq data supporting the findings of this study have been deposited in the Cancer Genome Atlas (TCGA) repository (TCGA-STAD). The other data are included within the article.

## Abbreviations

Gene: Full name
RECQL4: Homo sapiens RecQ protein-like 4
ATAD5: Homo sapiens ATPase family, AAA domain containing 5
GINS1: Homo sapiens GINS complex subunit 1 (Psf1 homolog)
ORC6: Homo sapiens origin recognition complex, subunit 6
RAD51: Homo sapiens RAD51-associated protein 2 (RAD51AP2)
EME1: Homo sapiens essential meiotic endonuclease 1
CCND1: Homo sapiens cyclin D1 (CCND1)
DBF4: Homo sapiens DBF4 homolog B (*S*. *cerevisiae*)
MCM2: Homo sapiens minichromosome maintenance complex component 2
NASP: Homo sapiens nuclear autoantigenic sperm protein (histone-binding)
EZH2: Homo sapiens enhancer of zeste homolog 2 (Drosophila) (EZH2)
PCNA: Homo sapiens proliferating cell nuclear antigen
CDC25A: Homo sapiens cell division cycle 25 homolog A
KRT8: Homo sapiens keratin 85
THOC6: Homo sapiens THO complex 6 homolog (Drosophila)
WT1: Homo sapiens SWT1 RNA endoribonuclease homolog
ATF2: Homo sapiens basic leucine zipper transcription factor, ATF-like 2
FOXP3: Homo sapiens forkhead box P3 (FOXP3)
HAT1: Homo sapiens histone acetyltransferase 1
NUF2: Homo sapiens NUF2, NDC80 kinetochore complex component, homolog
PLK1: Homo sapiens M-phase specific PLK1 interacting protein.
